# Antitumor effect of a WEE1 inhibitor and potentiation of olaparib sensitivity by DNA damage response modulation in triple-negative breast cancer

**DOI:** 10.1038/s41598-020-66018-5

**Published:** 2020-06-18

**Authors:** Dong-Hyeon Ha, Ahrum Min, Seongyeong Kim, Hyemin Jang, So Hyeon Kim, Hee-Jun Kim, Han Suk Ryu, Ja-Lok Ku, Kyung-Hun Lee, Seock-Ah Im

**Affiliations:** 1 Cancer Research Institute, Seoul National University, Seoul, Korea; 2 Biomedical Research Institute, Seoul National University Hospital, Seoul, Korea; 3 Department of Internal Medicine, Chung-Ang University College of Medicine, Seoul, Korea; 4 Department of Pathology, Seoul National University College of Medicine, Seoul, Korea; 5 Translational Medicine, Seoul National University College of Medicine, Seoul, Korea; 6 Department of Internal Medicine, Seoul National University College of Medicine, Seoul, Korea

**Keywords:** Breast cancer, DNA damage response

## Abstract

Due to its regulation of CDK1/2 phosphorylation, WEE1 plays essentially roles in the regulations of G2/M checkpoint and DNA damage response (DDR). WEE1 inhibition can increase genomic instability by inducing replication stress and G2/M checkpoint inactivation, which result in increased cellular sensitivity to DNA damaging agents. We considered an increase in genomic instability induced by WEE1 inhibition might be used to augment the effects of drugs targeting DNA repair protein. Typically, PARP inhibitors are effective in germline *BRCA 1/2* mutated breast and ovarian cancer, but their applicabilities in triple-negative breast cancer (TNBC) are limited. This study was conducted to investigate the anti-tumor effects of the WEE1 inhibitor, AZD1775, and the mechanism responsible for its potentiation of sensitivity to olaparib (a PARP inhibitor) via the modulation of DDR in TNBC cells. Our results suggest that AZD1775 could be used to broaden the application range of olaparib in TNBC and provide a rationale for a clinical trial of combined olaparib and AZD1775 therapy.

## Introduction

Triple-negative breast cancer (TNBC) is a breast cancer subtype that lacks estrogen receptor (ER) and progesterone receptor (PR) expression and does not exhibit human epidermal growth factor receptor 2 (HER2) amplification. TNBC accounts for 15–20% of all breast cancer cases and has more aggressive characteristics and higher rates of distant recurrence and shorter overall survivals than other breast cancer subtypes^1^. TNBC is also a heterogeneous disease with various subtypes, and as a result, translational studies based on the use of agents that target specific subtypes are being actively pursued^2^. However, the clinical applications of such agents are currently very limited, and thus, the systemic treatment of TNBCs is largely dependent on platinum containing, taxane, and anthracycline based chemotherapies. Unfortunately, durable responses to these treatments are limited by high resistance and recurrence rates and by adverse toxic effects. As a result, many research programs are being conducted to identify new targeting therapies effective in TNBC. As reported in the cancer genome atlas (TCGA) database, alterations of *RB* and *CCND1* are present in 22% of TNBC cases, and *TP53* mutations are detected in more than 80%^3^. Thus, dysregulation of the G1 cell cycle checkpoint is common in TNBC, and this results in higher mutation burdens because of high proliferation rates and replication stress accumulation observed at higher Ki-67 levels, which in turn, cause genomic instability^4^. Specifically, cell cycle checkpoint defects promote DNA replication and cell division, which result in damaged DNA accumulation and increase genetic instability^5^. These features have been proposed under the concept of synthetic lethality to inhibit other cell cycle checkpoints that were normally maintained, leading to cell death due to increased genetic instability caused by abnormal cell cycle progression.

WEE1 is a tyrosine kinase that inhibits the activation of CDK1 and CDK2, and thus, acts as a cell cycle regulator in the G2/M and S phases^6,7^. On the other hand, AZD1775 is a small molecular inhibitor of WEE1 and has been shown to cause cell cycle acceleration and apoptosis when applied with DNA damaging agents in various *TP53*-mutated cancers cell lines^8,9^. In addition, AZD1775 has been shown to be cytotoxic to cancer cells independently of the presence of TP53 mutation^10–12^, because activation of CDK1 promoted by WEE1 inhibition leads to early mitotic entry and mitotic catastrophe^13^. Furthermore, WEE1 also appears to be involved in replication fork stabilization and replication origin firing^14,15^. Recent studies have suggested that *Myc* amplification or *K-Ras* mutation, which can increase replication rates, may be sensitive markers of WEE1 inhibitor^16^. These results indicate WEE1 plays a role not only in the G2/M cell cycle phase but also S phase, and that it is strongly associated with genomic instability. However, the number of preclinical studies conducted on WEE1 is limited, and little information is available on its effects in aggressive TNBC subtypes with high replication rates, as reflected by high Ki-67 expression. Earlier studies on WEE1 inhibitors as monotherapies in breast cancer showed limited activities due to a lack of a clear understanding of the mechanisms responsible for their effects on cell cycle distribution.

In the case of homologous recombination repair deficient (HRD) cancers, PARP inhibitors offer a promising means of inducing synthetic lethality. The PARP inhibitors olaparib and talazoparib have been approved by the FDA as single agents for the treatment of metastatic breast cancer with the *BRCA1/2* (breast cancer 1/2) germline mutation. Sensitivity to PARP inhibitors is assessed using HRD, as reflected by germline and somatic *BRCA1/2* mutation statuses. However, inherited *BRCA1/2* mutations only account for ~5.3% of all breast cancers and <15% of TNBCs^3,17^. Recently, combinatorial strategies, including HRD induction therapy, have been proposed to expand the utilities of PARP inhibitors. Indeed, it has been reported that the antitumor effects of PARP inhibitors are enhanced when the HRD phenotype is induced by directly or indirectly regulating DNA repair molecules such as IGF1R, HDAC, ATR, or ATM inhibitors^18–21^. However, since IGF1R and HDAC inhibitors cannot be currently administered in breast cancer, a HRD induction strategy based on clinically applicable drugs is required. In this context, AZD1775 has also been reported to cause DNA damage accumulation and to increase sensitivity to DNA damaging agents^22^. Several clinical trials are currently being conducted on combinations of a WEE1 inhibitor and various DNA damaging agents, and some studies have done much to explain the role played by WEE1 in the DNA damage and repair pathways. In particular, it has been shown WEE1 regulates MUS81 nuclease activity by inhibiting CDK1 during the S phase, and that unstrained CDK1 activity caused by WEE1 inhibition leads to the unexpected activation of MUS81 and subsequent DNA fragmentation^15^, which provides a possible explanation of how WEE1 inhibition increases DNA damage. Others have argued WEE1 can regulate BRCA2-dependent homologous recombination repair (HR) via the CDK1 dependent phosphorylation of BRCA2^20^. Taken together, these observations and suggestions indicate WEE1 inhibition might induce the HRD phenotype. Based on these results, combinatorial PARP inhibitor or DNA damaging agent and WEE1 inhibitor treatments are being subjected to clinical trials. In particular, a clinical trial on combined treatment with olaparib and ATR inhibitor is being conducted in Phase II TNBC patients. However, few studies have evaluated how HR is regulated by WEE1 inhibition in BC. Therefore, we investigated the antitumor effects of a WEE1 inhibitor (AZD1775) and the mechanisms responsible for its effects on the cell cycle and DNA repair pathway as a monotherapy and in combination with a PARP inhibitor (olaparib), an ATR inhibitor (AZD6783), and a DNA damage-inducing agent (cisplatin) in six TNBC cell lines and in a Balb/c athymic nude mouse xenograft model. In addition, we explored the antitumor effects of AZD1775 and olaparib co-treatment in the presence or absence of BRCA mutations, and investigated how WEE1 inhibition influences RAD51-dependent HR in TNBC cell lines.

## Results

### AZD1775 induced apoptotic cell death in TNBC cells

**Figure 1:**
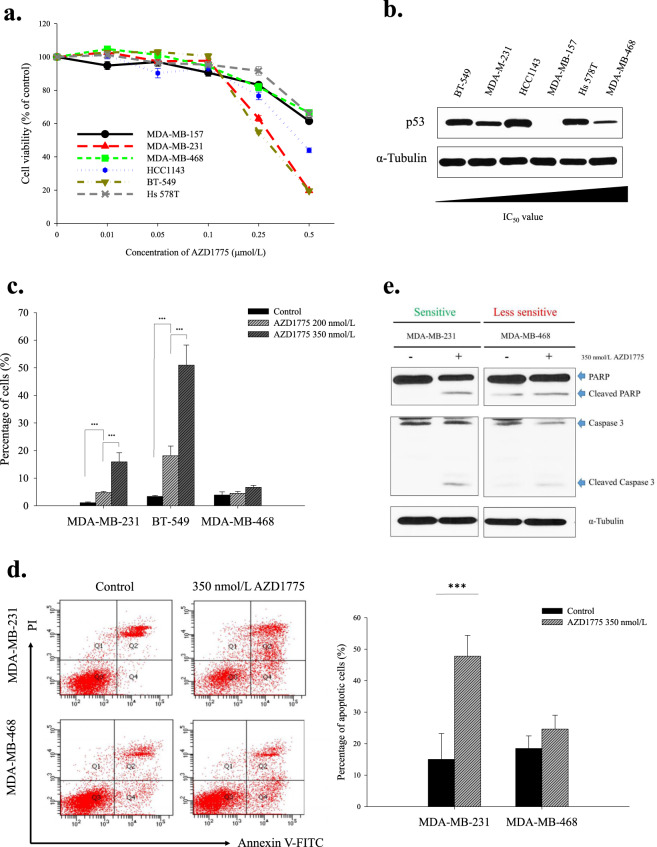
Anti-proliferative effect of AZD1775 in TNBC cells. (**a**) The different anti-proliferative effects of AZD1775 in TNBC cells. Growth inhibitions were measured using an MTT assay. Cells were treated with increasing doses of AZD1775 for 5 d. Percentages of surviving cells are presented with ±SE bars (*n* = 3). (**b**) p53 protein expression levels in TNBC cells increased with IC_50_ values. (**c**) AZD1775 treatment increased sub-G1 populations. Cellular DNA contents were measured by flow cytometry after PI staining. Sub-G1 proportions are shown in the bar graph. Bars represent ±SDs (*n* = 3). ***P < 0.001. (**d**) AZD1775 induced apoptosis in MDA-MB-231 cells. MDA-MB-231 and MDA-MB-468 cells were treated with AZD1775 at 350 nmol/L for 5 d, and annexin-V PI staining was performed to identify apoptotic cells. Bars represent ±SDs (*n* = 3). ***P < 0.001. (**e**) Caspase-dependent apoptosis induced by AZD1775. After 5 d of treatment with AZD1775 350 nmol/L, MDA-MB-231, and MDA-MB-468 cells were analyzed by Western blotting and probed with anti-PARP, caspase-3, and α-tubulin antibody. We used several gels to examine protein expression. But the results were all derived the same experiment, and the gels and blots were processed in parallel.

The anti-tumor effects of AZD1775 (WEE1 inhibitor) were assessed using an MTT assay in six TNBC cell lines (Fig. 1a). TNBC cells responded differently to AZD1775; IC_50_ values ranged from 0.36 to 0.81 µmol/L. MDA-MB-231 and BT-549 cells, which had IC_50_ values of <0.5 µmol/L were deemed sensitive, and MDA-MB-468 cells, which had an IC_50_ value of >0.5 were deemed moderately sensitive. According to previous report^23^, diminished phosphorylations of CDK1 and CDK2 (direct targets of WEE1) confirmed AZD1775 effectively downregulated target kinase activity (Supplementary Fig. S1a,b). In a previous study, the dependence of WEE1 inhibitor sensitivity on *TP53* mutation status was not resolved^22^, thus to investigate whether *TP53* mutation status was associated with WEE1 inhibitor sensitivity, we used the cancer cell line encyclopedia (CCLE) database to evaluate *TP53* mutation status. Sensitivity to AZD1775 varied among cell lines harboring a *TP53* hotspot mutation, and no relationship was observed between AZD1775 sensitivity and p53 protein level among the six TNBC cell-lines (Fig. 1a,b and Table 1). These observations demonstrated that the anti-proliferative effect of AZD1775 on TNBC cells was independent of the mutational status of *TP53*. Moreover, in sensitive cells, AZD1775 increased the proportion of cells in the sub-G1 phase, which is indicative of apoptosis (Fig. 1c). This was confirmed by Annexin V assays and by the detection of increased levels of cleaved PARP and caspase-3 in sensitive cells (Fig. 1d,e).

**Table 1:** IC_50_ values of AZD1775 in TNBC cells.

Cell lines	*TP53* mutation status	IC_50_ of AZD1775 (μmol/L)
MDA-MB-157	Mutant	0.5529
MDA-MB-231	Mutant	0.3550 ± 0.01
MDA-MB-468	Mutant	0.8151
HCC1143	Mutant	0.4538 ± 0.07
BT-549	Mutant	0.3381 ± 0.01
Hs 578 T	Mutant	0.7884

The TP53 mutation appeared in our TNBC cells, and the cells showed various IC50 values in response to the AZD1775 treatment, even when the TP53 mutation was present. IC50 values were calculated using SigmaPlot.

### AZD1775 induced aberrant the cell cycle in sensitive cells

**Figure 2:**
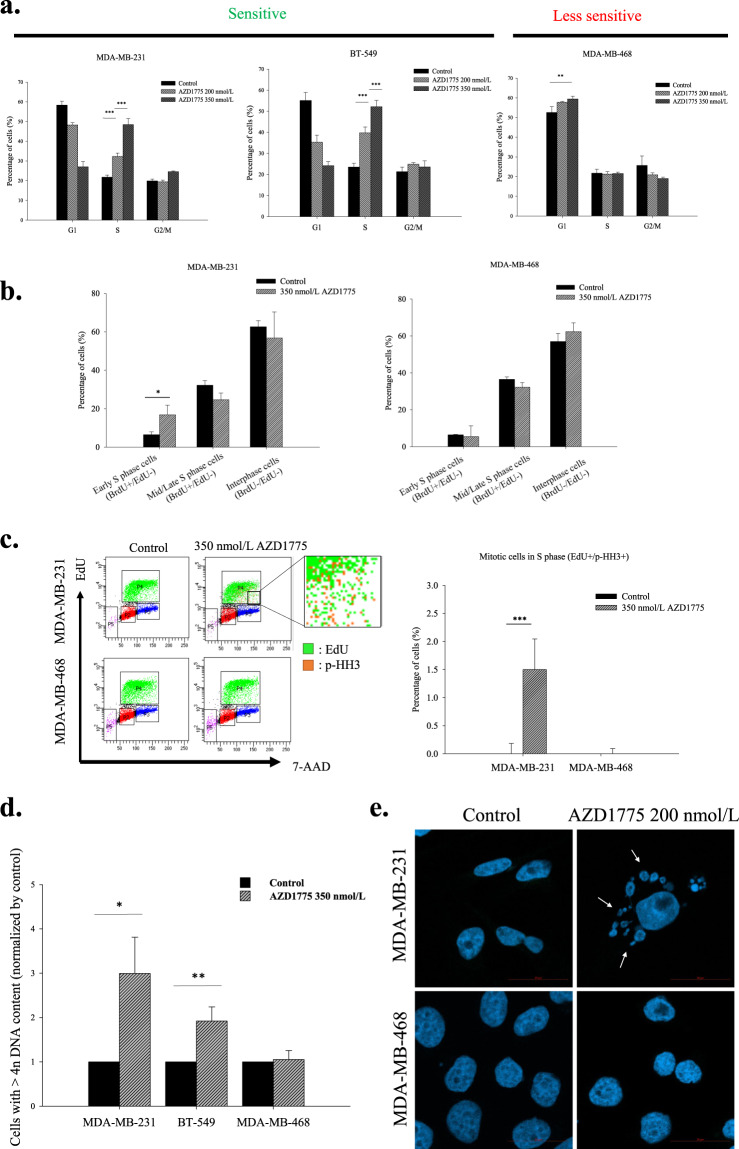
AZD1775 accelerated S phase progression and mitotic entry. (**a**) AZD1775 induced S phase accumulation in the MDA-MB-231 and BT-549 cell lines. TNBC cells were treated with AZD1775 at 350 nmol/L for 5 d, and cellular DNA contents were measured by flow cytometry after PI staining. The proportions of cells in each stage of the cell cycle are shown in the bar graph. Bars represent ±SDs (*n* = 3). **P < 0.01, ***P < 0.001. (**b**) AZD1775 accelerated S phase progression in MDA-MB-231 cells. Following treatment with 350 nmol/L AZD1775 for 24 h, S phase progression was examined by EdU-BrdU dual pulse labeling. EdU/BrdU(−/+) indicates the early S phase, and EdU/BrdU(+/+) indicates the mid/late S phase. At least 10000 cells were counted per group in an experiment. Data were collected from 3 independent experiments. Bars represent ±SDs. *P < 0.05. (**c**) Early mitotic entry induced by WEE1 inhibition in the MDA-MB-231 cell line. After treatment under the above conditions, p-HH3 (orange colored) antibody was applied to TNBC cells that had incorporated EdU (green colored) to investigate mitotic phase cells in S phase cells. At least 10000 cells were counted per group in an experiment. Data were collected from 3 independent experiments. Bars represent ±SDs. ***P < 0.001. (**d**) AZD1775 increased the proportion of cells with a DNA content of more than 4n. TNBC cells were treated with 350 nmol/L AZD1775 for 5 d and PI-stained. Cells with high DNA contents were identified by flow cytometry. Bars represent ±SDs (*n* = 3). *P < 0.05, **P < 0.01. (**e**) The proportion of multinucleated MDA-MB-231 cells increased in response to AZD1775. The nuclei of MDA-MB-231 and MDA-MB-468 cells were stained with DAPI, and the proportions of multinucleated cells were determined by confocal microscopy. White arrows indicate multinucleated nuclei. MDA-MB-231 and MDA-MB-468 cells were treated with 200 nmol/L AZD1775 for 5 d. A total of 100 cells per group were analyzed. Bars represent ±SDs (*n* = 3). **P < 0.01.

WEE1 is known to participate in the G2/M cell cycle checkpoint, and thus, cell cycle distributions were determined by FACS to investigate the effects of WEE1 inhibition on cell cycle progression. Proportions of cells in the S phase increased dose- and time-dependently in sensitive cell lines after AZD1775 treatment (Fig. 2a; Supplementary Fig. S2). To determine how AZD1775 influenced cell cycle progression in the S phase, two different thymidine analogs, that is, BrdU and EdU, were sequentially incorporated to investigate S phase progression (Fig. 2b). AZD1775 treatment increased the proportion of EdU/BrdU (−/+) cells (indicative of the early S phase), confirming AZD1775 accelerated initiation of the S phase. In addition, AZD1775 increased the proportion of cells expressing mitosis marker p-HH3 in EdU positive cells (indicative of the S phase) (Fig. 2c). Furthermore, long-term exposure of sensitive cells to AZD1775 significantly increased DNA contents to over 4n (Fig. 2d), indicating WEE1 inhibition resulted in abnormal mitotic exits and increased numbers of multinucleated cells (Fig. 2e), which is a characteristic of mitotic catastrophe^24^. Taken together, these results indicate AZD1775 influenced mitotic entry and exit as well as replication initiation and suggest its acts throughout the cell cycle.

### AZD1775 decreased double-strand DNA breaks repair capacity

**Figure 3:**
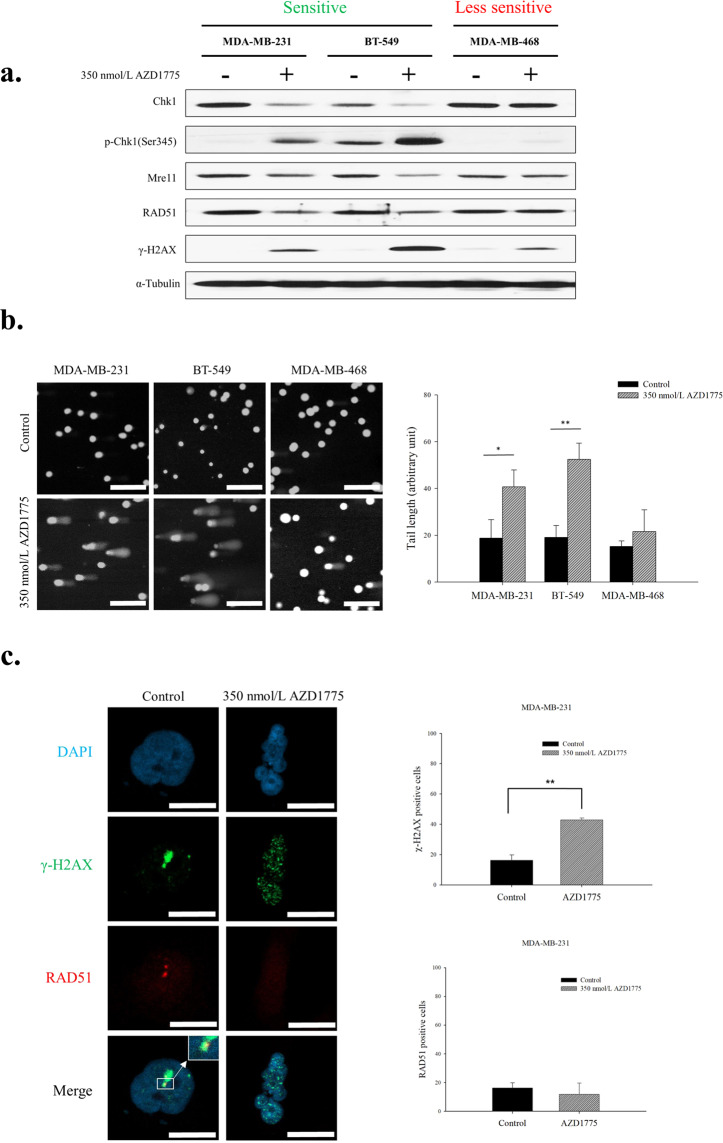
WEE1 inhibition resulted in HR impairment. (**a**) The expression levels of DNA damage response proteins were assessed by western blotting after treatment of AZD1775 350 nmol/L with MDA-MB-231, BT-549 and MDA-MB-468 for 48 h. (**b**) AZD1775 caused DNA damage accumulation. Cells were treated with 350 nmol/L AZD1775 for 5 d and DNA double-strand breaks in cells were assessed using a comet assay. Data presented are the mean tail length ±SD from a single representative experiment (*n* = 3). The scale bar indicates 200 µm. *P < 0.05, **P < 0.01. (**c**) AZD1775 inhibited the formation of RAD51 foci at sites of DNA damage. After treatment with 350 nmol/L of AZD1775 for 48 h, numbers of RAD51 (red) and γ-H2AX (green) foci in cells were determined by immunoblotting and confocal microscopy. A total of 100 cells per group were analyzed, and cells with at least 5 foci were regarded as positive. DNA was counterstained with DAPI (blue). The scale bar indicates 10 µm. Bars represent ±SDs (*n* = 3). **P < 0.01. We used several gels to examine protein expression. But the results were all derived the same experiment, and the gels and blots were processed in parallel.

Replication stress is one of the causes of DNA damage, and replicative stress and high genomic instability are characteristics of TNBC^4^. Furthermore, cell cycle disruption caused by WEE1 inhibition is likely to further increase replication stress and consequently increase the accumulation of damaged DNA. We found AZD1775 treatment resulted in Chk1 activation and elevated γ-H2AX levels in three TNBC cell-lines, regardless of AZD1775 response. However, the protein levels of involved in HR (RAD51, Mre11) were diminished in MDA-MB231 and BT-549 cells (Fig. 3a). Because the expression levels of DDR associated proteins were suppressed by AZD1775 treatment, we considered AZD1775 might modulate DDR activity, and thus, we used a comet assay to determine whether WEE1 inhibition caused DNA damage. Obvious tail lengthening indicated AZD1775 significantly increased DNA damage in the MDA-MB-231 and BT-549 cell-lines (Fig. 3b). Furthermore, these observations suggested AZD1775 increased DNA damage by depleting HR components. In addition, IFA assays consistently showed AZD1775 depleted DDR capacity. The increased levels of DNA damage in response to AZD1775 were confirmed by increased numbers of γ-H2AX foci, though the formation of RAD51 foci, which are involved in DNA strand exchange in HR, was inhibited by AZD1775 (Fig. 3c). These results demonstrate that AZD1775 induces DNA damage, and thus, aberrant cell cycle progression, and also reduces HR ability.

### Co-administration of olaparib and AZD1775 had a synergistic anti-proliferative effect in TNBC cells

**Figure 4:**
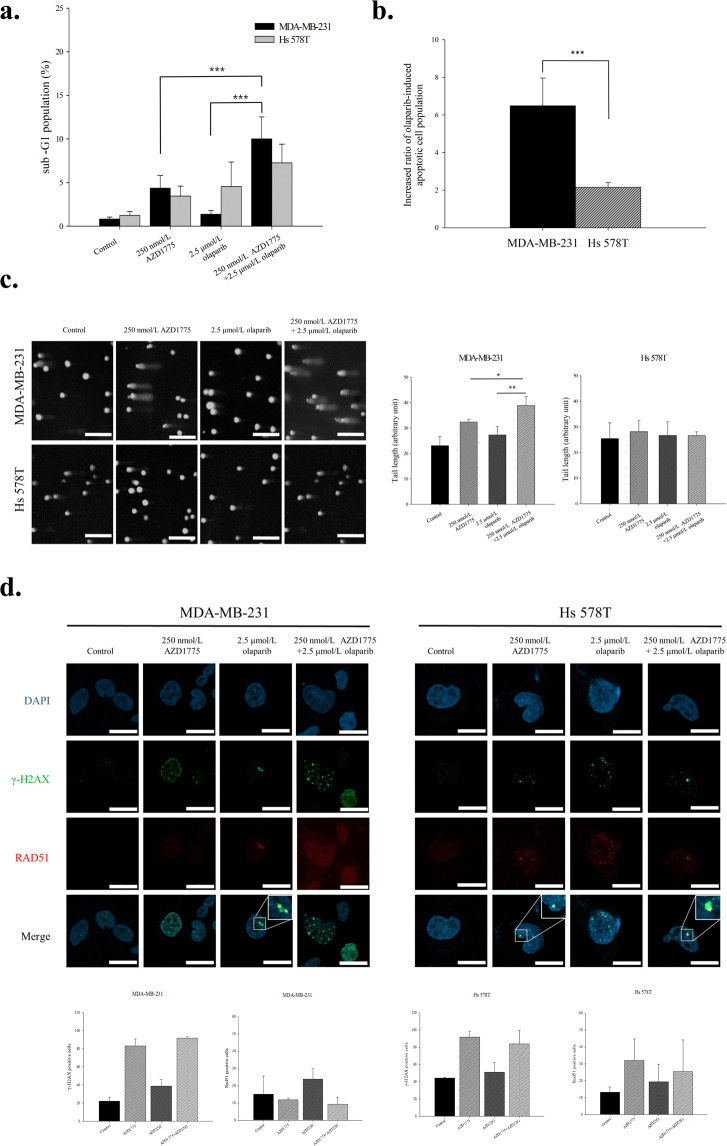
Co-administration of olaparib and AZD1775 had a synergistic, anti-proliferative effect on TNBC cells. (**a**) Sub-G1 population after co-treatment. MDA-MB-231 and Hs 578 T cells were treated with AD1775 at 250 nmol/L and/or olaparib at 2.5 μmol/L for 5 d, sub-G1 populations were investigated by flow cytometry. Bars represent ±SDs (*n* = 3). ***P < 0.001. (**b**) Increased sensitivity to olaparib by AZD1775. Cellular DNA contents were measured by flow cytometry after PI staining. Sub-G1 proportions are shown in the bar graph. Bars represent ±SDs (*n* = 3). ***P < 0.001. (**c**) Co-treatment with AZD1775 and olaparib increased damaged DNA accumulation as compared with treatment with either agent alone. MDA-MB-231 and Hs 578 T cells were treated with AD1775 at 250 nmol/L and/or olaparib at 2.5 μmol/L for 5 d. The scale bars indicate 200 μm. Data presented are the mean tail length ±SD from a single representative experiment. *P < 0.05, **P < 0.01. (one-way ANOVA: Duncan’s *post-hoc* Test). (**d**) AZD1775 inhibited the formation of RAD51 foci caused by olaparib. Blue indicates DAPI staining, representing DNA, green indicates γ-H2AX, representing the degree of DNA damage, and RAD51, which was used to confirm HR capacity, is indicated by red. The scale bar indicates 20 µm. 100 cells were counted per group in a treatment. Bars represent ±SDs (*n* = 3).

Olaparib is a target drug that can induce synthetic lethality in the presence of HR defects such as *BRCA1* or *BRCA2* germline mutation. We hypothesized that AZD1775 might increase sensitivity to olaparib by mimicking the BRCAness phenotype in TNBC cells. Combination indexes were used to determine whether combined treatment with olaparib and AZD1775 had a synergistic effect. Analysis revealed that the anti-proliferative effects of co-administration differed among TNBC cells (Table 2). Of the six cell lines, four (MDA-MB-157, MDA-MB-231, MDA-MB-468, BT-549) showed synergistic effects, whereas effects in two cell lines (HCC1143, Hs 578 T) effects were additive. The combination of AZD1775 and olaparib increased cell death based on the proportion of sub-G1 cells (Fig. 4a). Moreover, AZD1775 increased sensitivity to olaparib by more than 6-fold in MDA-MB-231 cells (Fig. 4b). To determine whether the DNA damage induced by olaparib was the result of HR depletion by AZD1775, we evaluated degrees of DNA damage after treating cells with AZD1775 and/or olaparib. Dual inhibition increased DNA damage accumulation as compared with mono-treatments (Fig. 4c). The reduction in DNA repair capacity induced by AZD1775 was investigated by examining RAD51 foci formation. In MDA-MB-231 cells RAD51 foci formation was not increased by AZD1775, whereas DNA damage was significantly increased. In contrast, in Hs 578 T cells, RAD51 foci formation increased as DNA damage increased (Fig. 4c). HRD assays were conducted to determine whether AZD1775 affected HR capacity. We found HR capacities were only meaningfully decreased by AZD1775 in MDA-MB-231 and BT-549 cells with a combination index of <0.8 (Supplementary Fig. S3). These findings suggest that AZD1775 can induce a HRD-like phenotype, and thus, enhance the anti-tumor effects of olaparib and probably those of other DNA damage-inducing agents like cisplatin and AZD6783 (Table S1).

**Table 2:** Combination index of AZD1775 with olaparib combination treatment.

Cell lines	IC_50_ of AZD1775 (μmol/L)	IC_50_ of Olaparib (μmol/L)	IC_50_ of AZD1775:Olaparib = 1:20 (μmol/L)	Combination index (ED_50_)
MDA-MB-157	0.5529	>10	0.3986	<0.8
MDA-MB-231	0.3550	>10	0.1963	<0.8
MDA-MB-468	0.7651	4.3013	0.0665	<0.8
HCC1143	0.4538	>10	0.4134	>0.8
BT-549	0.3381	>10	0.2587	<0.8
Hs 578 T	0.7884	>10	0.6053	>0.8

The concentration of AZD1775 was gradually increased from 0.1 to 1μmol/L for 5 d, and the AZD1775/olaparib dose ratio was 1:20. Cell growth inhibition was investigated by MTT assay and the combination index was measured using the Calcusyn software.

### Combined treatment with AZD1775 and olaparib significantly inhibited tumor growth in a xenograft model of MDA-MB-231 human breast cancer

**Figure 5:**
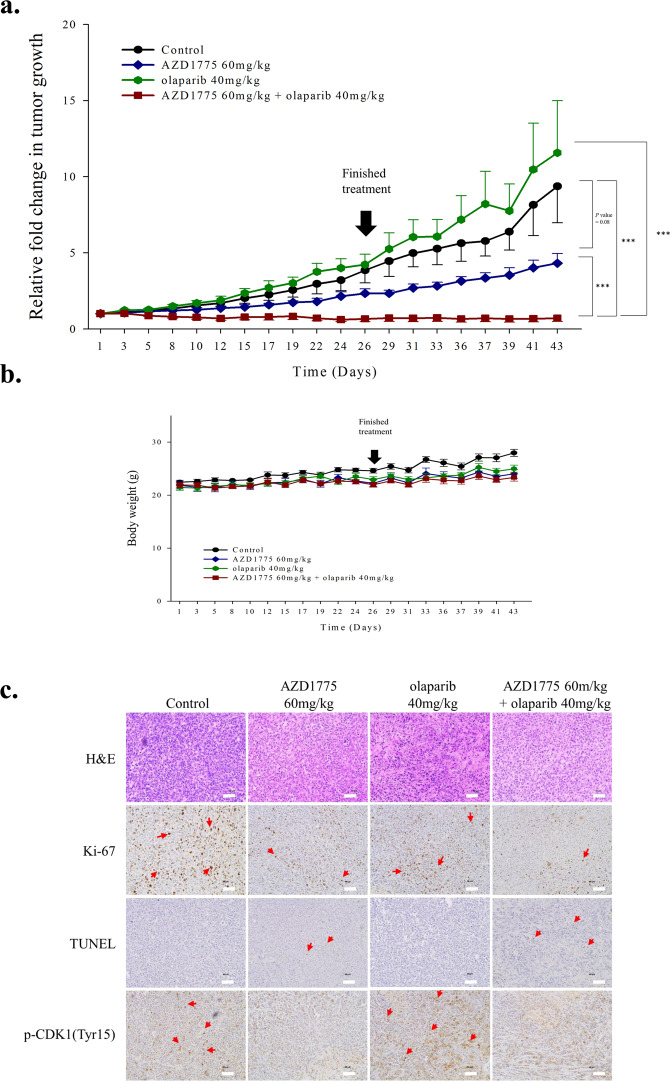
Co-treatment with AZD1775 and olaparib significantly inhibited tumor growth in our mouse xenograft model of MDA-MB-231 human breast cancer. (**a**) Treatment with AZD1775 plus olaparib significantly inhibited tumor growth the mouse model. Mice were treated with vehicle alone (*n* = 4), 60 mg/kg AZD1775 (*n* = 4), 40 mg/kg olaparib (*n* = 4), or both drugs (*n* = 5). Tumor volumes were measured three times weekly and are plotted with SE bars. ***P < 0.001. (**b**) Changes in mouse weights during the treatment period. Body weights were measured three times weekly. Bars, ± SEs. (**c**) Histological assessments of tumor response to AZD1775 alone and AZD1775 plus olaparib. The tumors were removed immediately after drug treatment, and pathologic examinations were conducted using hematoxylin and eosin (H&E) stained slides X20. Scale bars represent 50 μm. IHC staining for Ki-67 and a TUNEL assay were used to assess proliferation and apoptosis, respectively. AZD1775 reduced the level of p-CDK1(Tyr15). The scale bars represent 50 μm. Red arrows indicates each Ki-67, TUNEL, and p-CDK1(Tyr15) stained section.

To determine whether treatment with AZD1775 and olaparib has an anti-tumor effect *in vivo*, MDA-MB-231 cells were xenografted into Balb/c nude mice. Treatment with AZD1775 alone delayed tumor growth as compared with controls and a significant anti-tumor effect was observed in response to combined treatment (Fig. 5a and Supplementary Fig. S4). Moreover, treatment with ADZD1775 and/or olaparib maintained animal weights at a constant level, indicating that it was tolerated (Fig. 5b). Although quantification of Ki-67 and p-CDK1 levels in histological samples was limited by sample size and the extent of necrotic tissue, ki-67 levels were lower in tumor tissues of animals treated with AZD1775 plus olaparib than in animals treated with AZD1775 or olaparib and in those of vehicle controls, which indicated proliferative ability was reduced. Furthermore, this reduction in ki-67 levels was found to parallel apoptosis increases (as determined by TUNNEL assays). The kinase activity of WEE1 and the level of p-CDK1 (Tyr15) decreased in response to AZD1775 (Fig. 5c). Furthermore, combined treatment with AZD1775 and cisplatin also significantly and synergistically inhibited tumor growth (Supplementary Fig. S5a,b). These findings indicate that co-treatment with AZD1775 and olaparib has potential therapeutic benefits in TNBC.

## Discussion

Previous studies on WEE1 inhibitors have shown that G2/M abrogation by WEE1 inhibition leads to *TP53* mutation-dependent cytotoxicity, but we found no relation between *TP53* mutation and sensitivity to AZD1775 in TNBC cells in the present study. Other studies have reported that sensitivity to AZD1775 was not associated with *TP53* mutation, which concurs with our findings^10–12^. We also observed that WEE1 regulated the cell cycle from the S phase to mitotic exit. Sequential thymidine analog staining revealed that AZD1775 accelerated S phase progression and mitotic entry (Fig. 2b,c) and affected mitotic exit (Fig. 2d). AZD1775 treatment inhibits the expression of components of the spindle assembly checkpoint regulatory machinery such as BubR1 and Aurora kinase B (data not shown) and induces the development of multinucleated cells (Fig. 2e). These features are observed when cytokinesis is defective^25^. Indeed, it has been reported CDK1 is the main target of WEE1 inhibitor which is involved in cytokinesis^26–28^, and WEE1 inhibitors have been effectively applied to antimicrotubule cancer drugs (AMCDs)^29^. In fact, we observed that when WEE1 was suppressed in MDA-MB-231 and BT-549, DNA contents increased to 4n or more (Fig. 2d). Furthermore, increased levels of DNA damage induced by WEE1 inhibition paralleled degrees of genetic instability caused by cell cycle dysregulation in TNBC cells. In a previous study, DNA damage accumulation caused by WEE1 inhibition was determined to be dependent on MUS81 nuclease over-activation^14^, but co-activation of ERCC1, which is required for MUS81 dependent DNA fragmentation, was not observed in the present study (data not shown), although γ-H2AX expression was highly detected^30^. Therefore, we hypothesized that DNA damage accumulation following WEE1 inhibition occurred because of an increase in genomic instability caused by WEE1 inhibition and a failure to repair resulting damage sufficiently. We confirmed that WEE1 inhibition decreased the level of HR related proteins (Fig. 3a), which suggests WEE1 inhibition induces HRD by decreasing the ability of HR to repair cells. Indeed, co-treatment with AZD1775 and olaparib was found to be highly effective in BRCA proficient TNBC cells. We also found that AZD1775 enhanced sensitivities to cisplatin and an ATR inhibitor.

Recent phase II clinical trials have shown that AZD1775 enhances the efficacy of carboplatin in patients with *TP53*-mutated ovarian cancer refractory or resistant to platinum-based first-line therapy^31^. In addition, although fewer than 10% of patients harbored a BRCA mutation, indicating HRD involvement, a response rate of 43% was achieved. These results suggested that AZD1775 increased sensitivity to carboplatin by modulating HR. Indeed, several previous studies have shown AZD1775 increases responses to radiation^32,33^ and agents that cause DNA damage^8–10^. The present study confirmed the effects of AZD1775 on HR capacity and showed it inhibited the expression of HR machinery and disrupted DNA damage repair by inhibiting RAD51 foci formation.

Initially, we considered that HR inhibition by AZD1775 would increase sensitivity to PARP inhibitors by causing phenomena such as HRD. Furthermore, recent reports have revealed resistance to PARP inhibitors can be overcome by WEE1 inhibition^34,35^, which is consistent with our findings. Indeed, in the present study, co-administration of AZD1775 and olaparib increased cytotoxicity by suppressing DNA damage response and causing DNA damage accumulation. Our results suggest expansion of the HRD phenotype expands the applicability of olaparib, and that AZD1775 modulates the HR machinery and cell cycle regulation to suppress DNA repair and cause damaged DNA to accumulate. Furthermore, our findings support the use of WEE1 inhibition in combination with various other DNA damaging agents or DDR inhibitors, such as ATR inhibitors.

Previous studies have confirmed the inhibitory-phosphorylation of BRCA2 by CDK1 activation when WEE1 is inhibited. These results suggest WEE1 inhibition might lead to BRCA2 dependent HR deficiency and not to the regulation of BRCA2-independent HR. However, we found AZD1775 increased sensitivity to olaparib in BRCA proficient cells and in *BRCA 1/2* mutant cells such as HCC1937 and HCC1428, indicating AZD1775 induced BRCA2-independent HR modulation in TNBC cells. In our system, WEE1 inhibition decreased Mre11, Chk1, and RAD51 protein expressions and disrupted RAD51 localization to sites of DNA damage. This modulation of HR by WEE1 inhibition could result in DNA damage accumulation and increase sensitivity to PARP inhibitors in TNBC cells, regardless of BRCA mutation status. Although we found that AZD1775 prevented RAD51 foci formation in TNBCs, we suggest the mechanism be investigated further.

Clinical trials are currently being undertaken on AZD1775 and olaparib co-treatment in TNBC, based in part on the results of our preclinical experiments. Two clinical trials involving induction of the HRD phenotype are ongoing in patients with genetic alterations of genes associated with a high proliferation rate or genetic instability. Furthermore, evidence indicates that combined treatment with WEE1 and PARP inhibitor might only be effective in HRD cancers^36–39^. However, with the exception of HRD markers, germline BRCA mutations in breast cancer remain to be identified, and the population of patients with HRD and a BRCA mutation is limited. Therefore, a strategy of widening the HRD population by WEE1 inhibition might help overcome the clinical limitations of PARP inhibitors.

In conclusion, we describe the mode of action of AZD1775 alone and of AZD1775 plus olaparib in TNBC cells. Our results indicate that co-treatment with PARP and WEE1 inhibitors might facilitate the development of clinical trial strategies for TNBC.

## Materials and methods

### Reagents

AZD1775, a WEE1 inhibitor, and olaparib, a PARP inhibitor, and AZD6783 a ATR inhibitor were provided by AstraZeneca (Macclesfield, Cheshire, UK) and dissolved in dimethyl sulfoxide (DMSO) at 10 mmol/L to produce stock solutions.

### Cell line and culture

Human breast cancer cells (MDA-MB-157, MDA-MB-231, MDA-MB-468, HCC1143, BT-549, Hs 578 T) were purchased from the American Type Culture Collection (ATCC, Manassas, VA, USA) and authenticated by short tandem repeat analysis. During storage, cultures were maintained in a 5% CO_2_ humidified atmosphere at 37 °C in RPMI-1640 (Welgene, Inc., Daegu, Korea) supplemented with 10% FBS (GIBCO, Thermo Fisher Scientific Inc., Waltham, MA, USA) and 10 µg/mL gentamicin (Cellgro, Manassas, VA, USA).

### Cell growth inhibition assay

Cell viabilities were determined using an MTT assay, as previously described^40^. Briefly, cells were seeded at a density of 3–8 × 10^3^ cells per well in 96-well plates and incubated overnight at 37 °C and treated with AZD1775 or olaparib alone or in combination with AZD1775 and olaparib at different concentrations for 5 d. Following treatments, MTT solution was added to each well, plates were incubated for 4 h at 37 °C, medium was removed, and formazan crystals were dissolved in DMSO. Cell viabilities was evaluated by measuring the well absorbances at 540 nm using a VersaMax™ microplate reader (Molecular Devices, Sunnyvale, CA, USA). The combined effects of AZD1775 and olaparib were assessed using Calcusyn software (Biosoft, Cambridge, UK). Combination indexes (CI), which were used to evaluate the effects of two-drug combinations, were calculated using the Chou-Talalay method^41^. Drug synergism was defined as a CI value of <0.8, while antagonism was defined as a value of >1. Additivity was defined as a CI value of >0.9 to <1.

### Western blot analysis

Cells were collected after treatments, washed with ice-cold PBS, and incubated in extraction buffer (50 mM Tris-Cl (pH 7.4), 150 mmol/L NaCl, 1% NP40, 0.1% sodium deoxycholate, 0.1% sodium dodecyl sulfate (SDS), 50 mM sodium fluoride, 1 mM sodium pyrophosphate, 2 mM phenylmethylsulfonyl fluoride, 1 mg/mL pepstatin A, 0.2 mmol/L leupeptin, 10 μg/mL aprotinin, 1 mM sodium vanadate, 1 mM nitrophenyl phosphate, and 5 mmol/L benzamidine) on ice for 30 min. Lysates were cleared by centrifugation at 13,000 rpm for 20 min, and equal amounts of proteins were separated on 8%–15% SDS-polyacrylamide gels. Resolved proteins were transferred to nitrocellulose membranes and blots were probed with primary antibodies overnight at 4°C. Antibodies against p53(cat.no.126), CDK1(cat.no.54), CDK2(cat.no.163), WEE1(cat.no.325), RAD51(cat.no.377467), and Chk1(cat.no.7898) were obtained from Santa Cruz Biotechnology (Santa Cruz, CA, USA). Antibodies against caspase 3(cat.no.9662), p-CDK1(Tyr15)(cat.no. 9111), p-WEE1(Ser642)(cat.no.4910), MYT1(cat.no.4282), p-Chk1(Ser345) (cat.no.2348), and Mre11(cat.no.4847) were purchased from Cell Signaling Technology (Beverley, MA, USA). Anti-phosphorylated histone H2AX(clone JBW301)(cat.no.03-636) was supplied by Millipore (Billerica, MA, USA), anti-PARP(cat.no.556494) by BD Biosciences (Bedford, MA, USA), and α-tubulin antibody(cat.no.5168) (the control) by Sigma Aldrich (St. Louis, MO, USA). Antibody binding was detected using an enhanced chemiluminescence system, according to the manufacturer’s instructions (Amersham Biosciences; Piscataway, NJ, USA).

### Cell cycle analysis

Cells treated with AZD1775 and/or olaparib were harvested, fixed in 70% ethanol, and stored at −20 °C. After 48 h they were dissolved in 10 μg/mL RNase A (Sigma Aldrich) at 37 °C for 20 minutes, treated with 20 μg/mL propidium iodide (Sigma Aldrich), and their DNA contents (10,000 cells per group) were determined using a FACS Canto II flow cytometer (BD Biosciences).

### Apoptosis assay

After cells had been exposed to AZD1775, degrees of apoptosis were investigated using the Annexin V/PI binding assay according to the manufacturer’s instructions (BD Bioscience). Briefly, harvested cell suspensions were incubated with Annexin V for 15 minutes at room temperature in the dark and then PI stained for 5 minutes at room temperature. Sample fluorescences were determined by flow cytometry (BD Biosciences). At least 10000 cells were counted per sample.

### Edu-BrdU dual pulse labeling

The level of incorporation of EdU and BrdU determined the progress of S phase, Click-iT EdU Flow Cytometry Assay kits (Thermo Fisher Scientific Inc) and FITC-conjugated anti-BrdU (BD Biosciences) were used. Before being harvested, cells were treated with 10 mmol/L of EdU for 1 h, 20 mmol/L BrdU for 15 min, fixed in 70% ethanol, and stored at −20 °C. DNA denaturation was performed in 4 M HCl for 20 min at room temperature, after which cells were resuspended in phosphate/citric acid Alternatively, BrdU and EdU incorporation levels were determined using S phase, using kit and antibody buffer. Cells were then treated with 7-AAD (BD Biosciences) and fluorescence was measured using a FACS Canto II flow cytometer (BD Biosciences). Finally, pacific blue-conjugated phospho-histone H3 (Ser10) and Click-iT EdU Flow Cytometry Assay kits (Thermo Fisher Scientific Inc) were used to identify mitotic entry status.

### Comet assay

Alkaline comet assays were conducted using a Trevigen Comet Assay kit (Trevigen, Gaithersburg, MD, USA) according to the manufacturer’s instructions. Incorporated sybr-green was detected using a Zeiss LSM 800 laser scanning microscope. Photographs were taken at a magnification of X20, and tail intensities were measured using the Comet assay IV program (Andor Technology, Belfast, UK).

### Immunofluorescence assay

Cells were plated on poly-L-lysine coated coverslips and 48 h later, treated with AZD1775 and/or olaparib for 2 d, fixed in 3.7% paraformaldehyde, and permeabilized with 0.5% Triton X-100 in PBS (PBS-T). Cells were then treated with anti-RAD51 (cat.no.377467) and anti-γ-H2AX (cat.no.03-636), rinsed three times for 10 minutes in PBS, incubated with appropriate fluorophore-conjugated secondary antibodies (Invitrogen, Carlsbad, CA, USA), and counterstained with DAPI (500 nmol/L, Invitrogen). Coverslips were then mounted onto slides using Faramount aqueous mounting medium (Dako, Glostrup, Denmark). Immunofluorescence was visualized using a Zeiss LSM 800 laser scanning microscope and photographs were taken at a magnification of X40.

### Homologous recombination repair deficiency assay

Cells were transfected with 10 µg of DR-GFP cDNA (Addgene, MA, USA) using lipofectamine, and 24 h later, cells with chromosomally integrated constructs were selected by adding 1 mg/mL G-418 (Takara Bio USA; Mountain View, CA, USA) and 0.75 µg/mL puromycin (Sigma) for 2 days. DR-GFP cDNA transfected cells were then transfected with 5 µg I-Sce1 plasmid (Addgene) using lipofectamine for 2 h, treated with AZD1775 for 2 d, and trypsinized. GFP-contents in live cells were determined by flow cytometry.

### ***In vivo*study**

To measure the *in vivo* activities of AZD1775 and/or olaparib and cisplatin, 38 female Balb/c athymic nude (5-wk-old) were purchased from Central Lab Animal Inc. (Seoul). MDA-MB-231 cells (1 × 10^8^) were subcutaneously injected into the right flank of each mouse. When tumor volumes reached 150 to 200 mm^3^, mice were randomly assigned different treatments (five per group), that is, to receive vehicle, AZD1775, olaparib, or AZD1775 + olaparib. Animals were administered AZD1775 at 30 or 60 mg/kg and olaparib at 40 mg/kg via oral gavage once daily for 28 consecutive days. When required cisplatin 4 mg/kg was administered by intraperitoneal injection twice weekly during the 4-week treatment period. Tumor volumes were measured three times a week during the experimental period and calculated using the following formula: (width^2^ × height)/2. At the end of the experimental period on day 29, mice were euthanized by CO_2_ insufflation and tumors were excised for further analysis. All animal experiments were approved by Seoul National University Institutional Animal Care and Use Committee (IACUC) (Seoul) and were conducted in accordance with institutional guidelines required by Seoul National University (Seoul) (SNU-151224-1-1).

### Immunohistochemistry

Histologic sections from individual paraffin-embedded xenograft tumor tissues were deparaffinized and rehydrated. IHC detection of proliferating cells was conducted using anti-rabbit polyclonal antibody against Ki-67 (Genetax Inc, CA, USA) at a dilution of 1:100. Apoptosis was detected using the TUNEL assay-based ApopTag *In situ* Apoptosis Detection Kit (Thermo Fisher Scientific Inc). p-CDK1 (Tyr15 residue) antibody (cat.no. P00209) (Boster Biological Technology, CA, USA) was diluted at 1:50.

### Statistical analysis

The two-sided Student’s *t*-test and one-way ANOVA, followed by Duncan’s multiple range test in SigmaPlot version 14.0 (Systat Software Inc., San Jose, CA, USA) was used to determine the significances of differences. Results are presented as means ± standard errors (SEs) or standard deviation (SD), and *P*-values of <0.05 were considered statistically significant and *P*-values of <0.1 were considered meaningful.

## Supplementary information

Supplementary information
