# Supplementary material for: Antitumor effect of a WEE1 inhibitor and potentiation of olaparib sensitivity by DNA damage response modulation in triple-negative breast cancer
Source: Sci Rep. 2020 Jun 18;10:9930. doi: 10.1038/s41598-020-66018-5 (PMC7303169; doi:10.1038/s41598-020-66018-5)
Supplement: Supplementary file 1 — Supplementary information [file 41598_2020_66018_MOESM1_ESM.pdf]

Antitumor effect of a WEE1 inhibitor and potentiation of olaparib sensitivity by DNA damage response modulation in triple-negative breast cancer.

Dong-Hyeon Ha[1], Ahrum Min[1,2], Seongyeong Kim[1], Hyemin Jang[1,2], So Hyeon Kim[1], Hee-Jun Kim [1,2,3], Han Suk Ryu[4], Ja-Lok Ku[1], Kyung-Hun Lee[1,2,5,6], Seock-Ah Im[1,2,5,6]*

[1]*Cancer Research Institute, Seoul National University, Seoul, Korea*

[2]*Biomedical Research Institute, Seoul National University Hospital, Seoul, Korea*

[3]*Department of Internal Medicine, Chung-Ang University College of Medicine, Seoul, Korea*

[4]*Department of Pathology, Seoul National University College of Medicine, Seoul, Korea*

[5]*Translational Medicine, Seoul National University College of Medicine, Seoul, Korea*

[6]*Department of Internal Medicine, Seoul National University College of Medicine, Seoul, Korea*

***Corresponding author:**

Seock-Ah Im, M.D., Ph.D.

Department of Internal Medicine

Seoul National University College of Medicine

101 Daehak-ro, Jongno-gu, Seoul, 03080, Korea

Tel: + 82-2-2072-0850

Fax: 82-2-762-9662

E-mail: moisa@snu.ac.kr

Supplementary Figure S1

a.

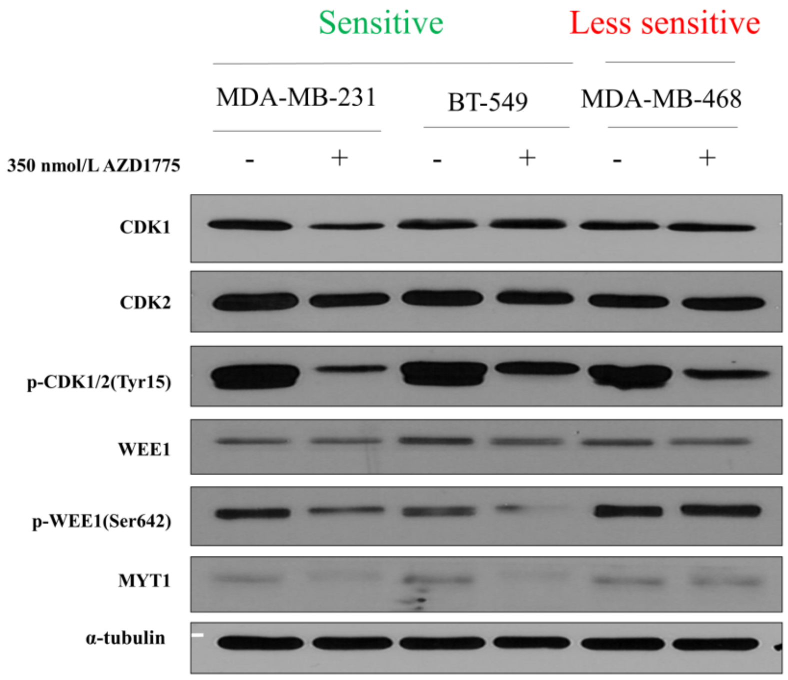

b.

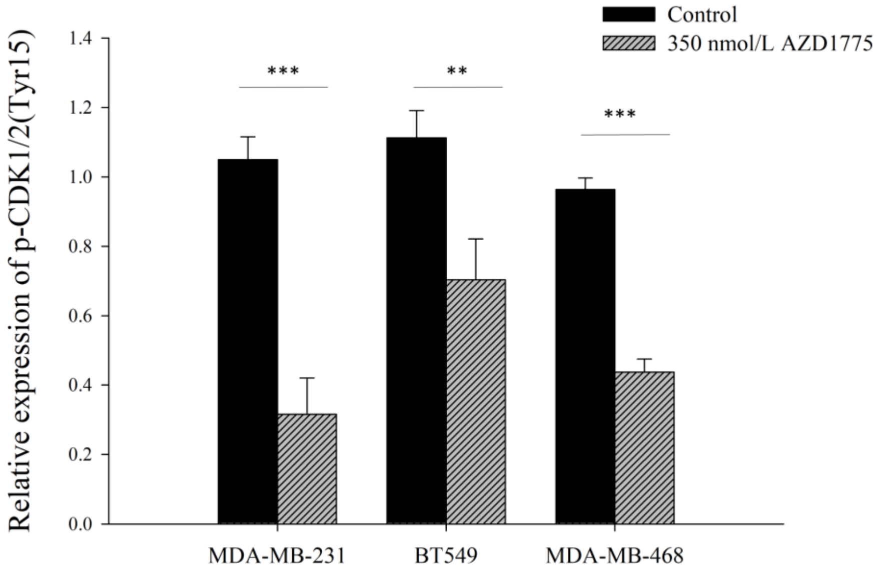

Supplementary Figure S2

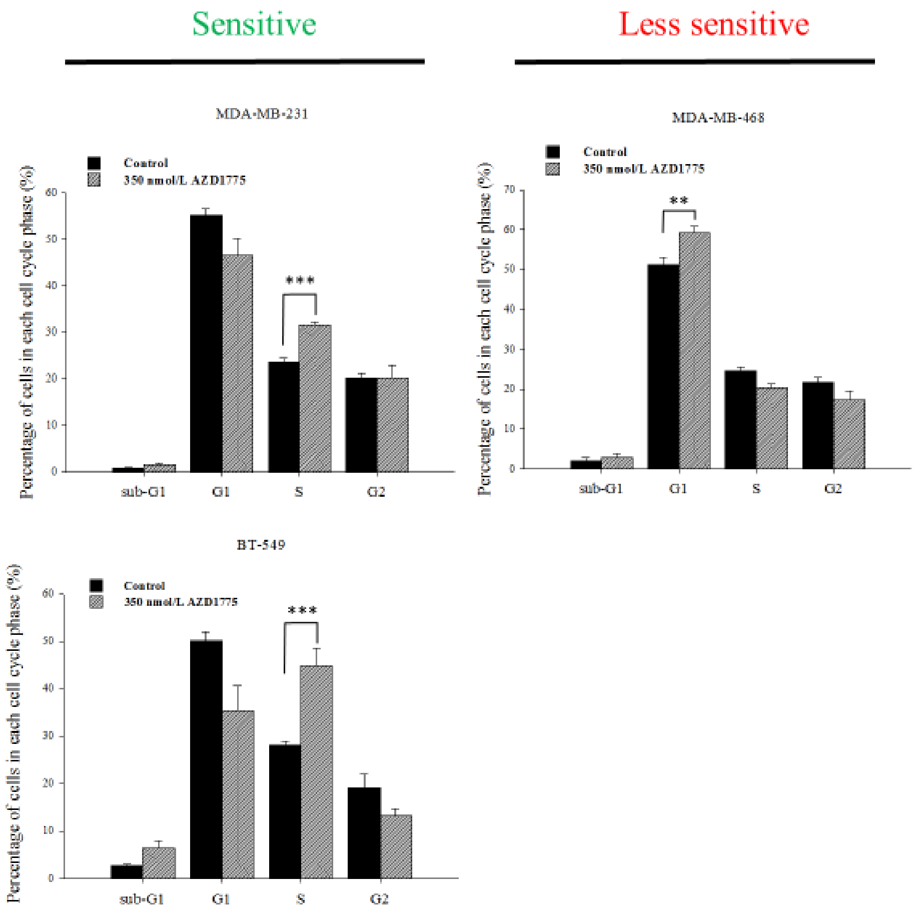

Supplementary Figure S3

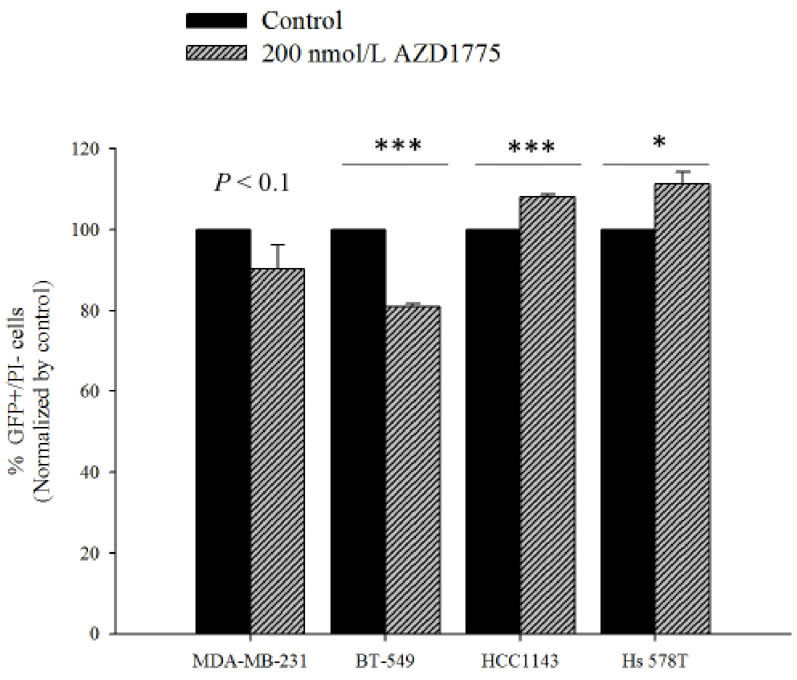

Supplementary Figure S4

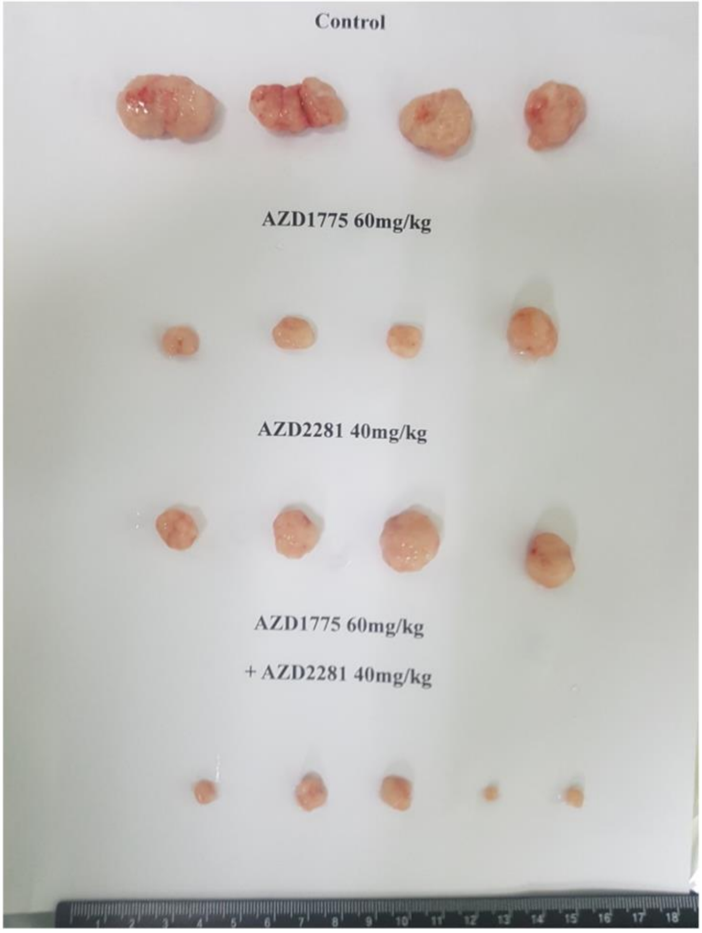

Supplementary Figure S5

a.

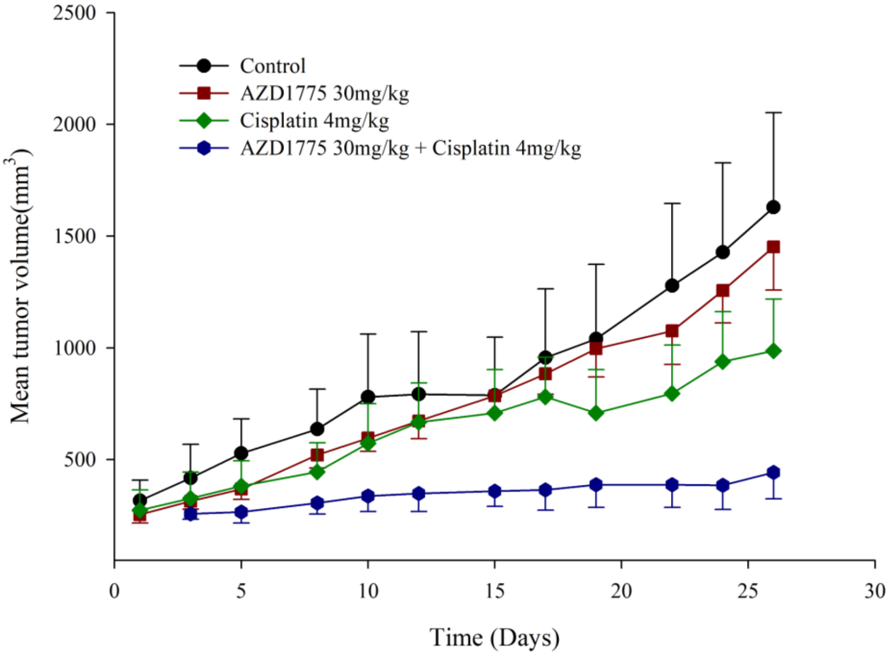

b.

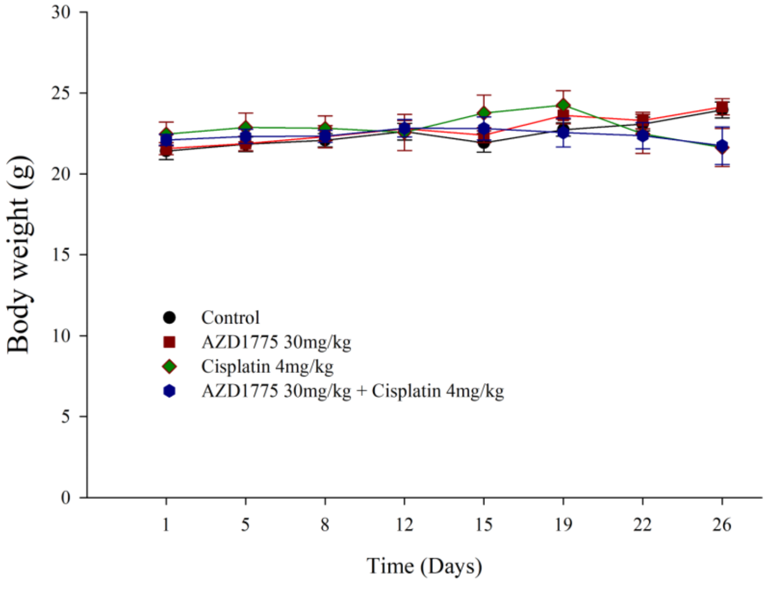

Supplementary Table S1

Cell lines	*TP53* mutation status	Combination index(ED_{50}) of Cisplatin	Combination index(ED_{50}) of ATR inhibitor
MDA-MB-157	P88fsX52	0.34	0.3
MDA-MB-231	R280K	0.73	0.2
MDA-MB-468	R273H	0.16	1.1
BT-549	R249S	0.79	1.0
Hs578T	V157F	0.62	>1

Supplementary Figure Legends

Figure S1. Kinase activity of WEE1 was inhibited by AZD1775. (a). The expression levels of each protein in TNBC cells were measured by western blotting after 350 nmol/L AZD1775 treatment for 5 d. MYT1 is known to play a compensatory role in WEE1. α-tubulin was used as a loading control. (**b**). Phosphorylation of the Tyr15 residue of CDK1 was normalized using Image J. We used several gels to examine protein expression. But the results were all derived the same experiment, and the gels and blots were processed in parallel. Bars, ±SD (n = 3). **P<0.01. ***P<0.001.

Figure S2. Cell cycle distribution following AZD1775 treatment at an early time point. After the indicated dose of AZD1775 was applied for 24 h, the proportion of cells in different phases of the cell cycle was confirmed by FACS. Bars, ±SD (n = 3). **P<0.01. ***P<0.001.

Figure S3. Decreased repair capacity by WEE1 inhibitor. GFP positive cells were counted in PI-negative cells. Normalization is based on the control values of the counted cell. Bars, ±SD. (n = 3). *P<0.05, ***P<0.001.

Figure S4. Co-treatment with AZD1775 and olaparib significantly inhibited tumor growth. Image of tumor tissues following MDA-MB-231 cell derived xenograft model in nude mice at 43 days. Mice were treated with vehicle alone (n=4), 60 mg/kg AZD1775 (n=4), 40 mg/kg olaparib (n=4), or both drugs (n=5).

Figure S5. Combination treatment of cisplatin and AZD1775 showed a synergistic anti-tumor effect *in vivo* in an MDA-MB-231 xenografted mouse model. (**a**). The MDA-MB-231 human breast cancer cell line was xenografted to more than four mice per group. The mice were treated with vehicle

alone (n=4), 40 mg/kg AZD1775 (n=4), cisplatin 4 mg/kg (n=4) and both drugs in combination (n=5). AZD1775 plus cisplatin significantly inhibited tumor growth. Tumor volumes were measured three times weekly and are plotted with ±SE bars. (**b**). The toxicity of the drug was determined by measuring changes in the weight of the mouse. Bars, ± SEs.

Table S1. Combined treatment of AZD1775 with cisplatin or ATR inhibitor AZD6738 shows a synergistic anti-tumor effect in TNBC cells. The concentration of AZD1775 was gradually increased from 0.1 to 1μmol/L for 5 d. The AZD1775/cisplatin and AZD1775/ATR inhibitor AZD6738 dose ratio was 1:10. Cell growth inhibition was investigated by MTT assay and the combination index was measured using the Calcusyn software.
